# Visualizing band offsets and edge states in bilayer–monolayer transition metal dichalcogenides lateral heterojunction

**DOI:** 10.1038/ncomms10349

**Published:** 2016-01-18

**Authors:** Chendong Zhang, Yuxuan Chen, Jing-Kai Huang, Xianxin Wu, Lain-Jong Li, Wang Yao, Jerry Tersoff, Chih-Kang Shih

**Affiliations:** 1 Department of Physics, University of Texas at Austin, Austin, Texas 78712, USA; 2 Physical Sciences and Engineering Division, King Abdullah University of Science and Technology, Thuwal 23955-6900, Kingdom of Saudi Arabia; 3 Department of Physics and Center of Theoretical and Computational Physics, University of Hong Kong, Hong Kong, China; 4 Institute of Physics, Chinese Academy of Sciences, Beijing 100190, China; 5 IBM Research Division, T.J. Watson Research Center, Yorktown Heights, New York 10598, USA

## Abstract

Semiconductor heterostructures are fundamental building blocks for many important device applications. The emergence of two-dimensional semiconductors opens up a new realm for creating heterostructures. As the bandgaps of transition metal dichalcogenides thin films have sensitive layer dependence, it is natural to create lateral heterojunctions (HJs) using the same materials with different thicknesses. Here we show the real space image of electronic structures across the bilayer–monolayer interface in MoSe_2_ and WSe_2_, using scanning tunnelling microscopy and spectroscopy. Most bilayer–monolayer HJs are found to have a zig-zag-orientated interface, and the band alignment of such atomically sharp HJs is of type-I with a well-defined interface mode that acts as a narrower-gap quantum wire. The ability to utilize such commonly existing thickness terraces as lateral HJs is a crucial addition to the tool set for device applications based on atomically thin transition metal dichalcogenides, with the advantage of easy and flexible implementation.

## Abstract (web summary)

Heterojunctions of two-dimensional materials are used to design electronic and optoelectronic devices. Here, the authors show that zigzag terraces between monolayers and bilayers form atomically sharp type-I heterojunctions, resulting in a wire-like interface both in WSe_2_ and in MoSe_2_.

## Graphical abstract

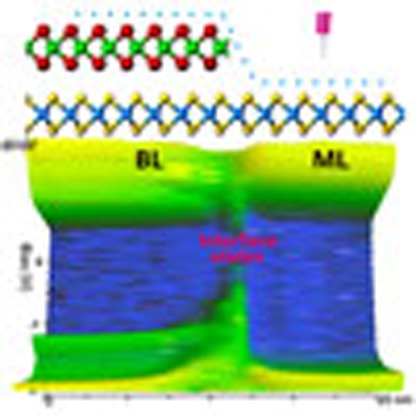

A heterojunction (HJ) is an interface between two different semiconductors. The difference in the electronic structures of the two materials results in potential discontinuities at the interface for electrons (conduction band offset) and holes (valence band offset). The introduction of heterostructures^1^ has enabled new types of electronic and photonic devices, transforming semiconductor technology. The recent emergence of two-dimensional (2D) semiconductors creates exciting new opportunities to push semiconductor heterostructures towards a new frontier^2^,^3^,^4^,^5^,^6^,^7^.

Vertically stacked van der Waals heterostructures have been quickly recognized as a powerful platform to create atomically thin heterostructures with great design flexibility^2^,^8^,^9^,^10^. Indeed, van der Waals heterostructures have been realized using different combinations of 2D materials, including transition metal dichalcogenides (TMDs), graphene and boron nitride^11^,^12^,^13^,^14^,^15^. Interesting properties, such as the observation of interface excitons^16^ and the determination of band alignments have been demonstrated recently in TMDs vertical heterostructures^17^. Less attention has gone to the possibility of creating ‘lateral HJs', where the junction is now a line interface between two 2D materials, reducing the dimensionality of heterostructures even further. Using direct chemical vapour deposition (CVD) growth, lateral junctions between different TMDs have been recently demonstrated^18^,^19^,^20^,^21^,^22^. However, theoretical calculations suggest that the heterostructures formed between the four most common TMD compounds (M=Mo, W; X=S, Se) all have type-II band alignment^23^,^24^, and the equally (if not more) important type-I HJs are still missing. How to create a lateral type-I HJ with an atomically sharp and straight interface, and characterize its band profile at the atomic scale, remain as significant challenges to overcome in order to advance this new frontier based on 2D semiconductors.

Previous investigations have shown that the bandgaps of TMD films greatly depend on the number of layers^25^,^26^. So one may naturally ask: can we create a well-defined lateral HJ between regions of different thicknesses, for example, a bilayer–monolayer interface? If so, is such a HJ type-I or type-II? Moreover, as the junction interface is also a step edge, how does this step edge influence the electronic structures of the HJ?

Here we study this new type of lateral heterostructures between bilayer (BL) and monolayer (ML) TMDs using low-temperature scanning tunnelling microcopy and spectroscopy (STM/S). We first show that atomically sharp and smooth interfaces with the desired type-I band alignment can indeed be formed. Moreover, we discover the presence of interface states with a narrow gap that act as interface quantum wires. Because the junction is formed between the direct gap monolayer TMD and the indirect gap bilayer TMD, the band edge carriers on the two sides of the junction are from different momentum space regions. This feature, coupled with the ability to control the interface through the edge states, offers the opportunity for novel device concepts in reduced dimensions.

## Results

### WSe_2_ bilayer–monolayer HJs

**Figure 1:**
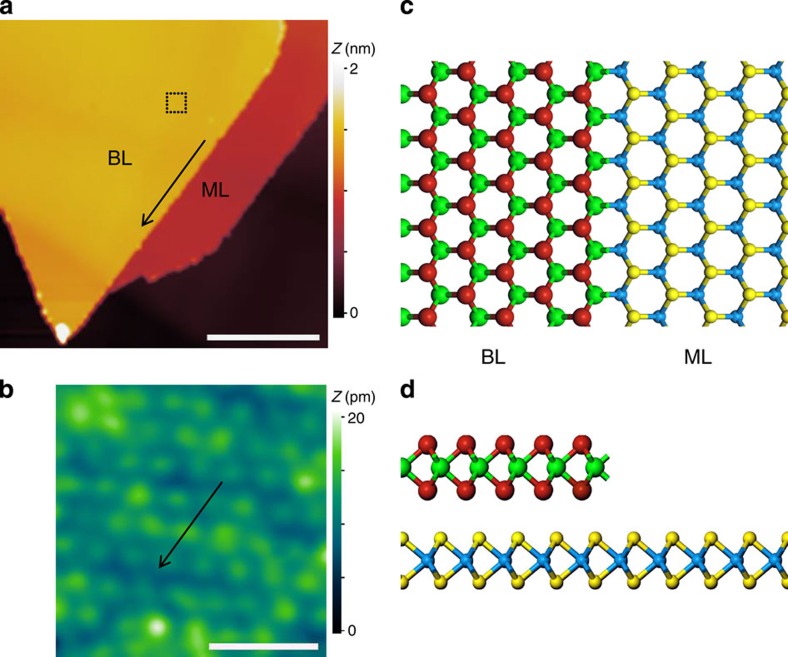
STM images and schematic models of BL–ML TMD lateral heterojunctions. (**a**) STM image for a naturally formed BL–ML WSe_2_ HJ grown on HOPG by chemical vapour deposition. The bilayer and monolayer regions are labelled as shown. (**b**) Atomic resolution image taken on bilayer region where a dash square is labelled in **a** (not to scale). The sample biases and tunnelling currents used are (**a**) 3 V, 8 pA, (**b**) −1.0 V, 10 pA. The arrows in **a** and **b** indicate the zig-zag orientation of the bilayer–monolayer interface. (**c,d**) Schematic models of the BL–ML heterojunction viewing from top and side, respectively. The green and cyan represent the metal atoms in second and first layer respectively, while the red and yellow corresponding for chalcogen atoms. Scale bar, 100 nm (**a**); 1 nm (**b**).

**Figure 2:**
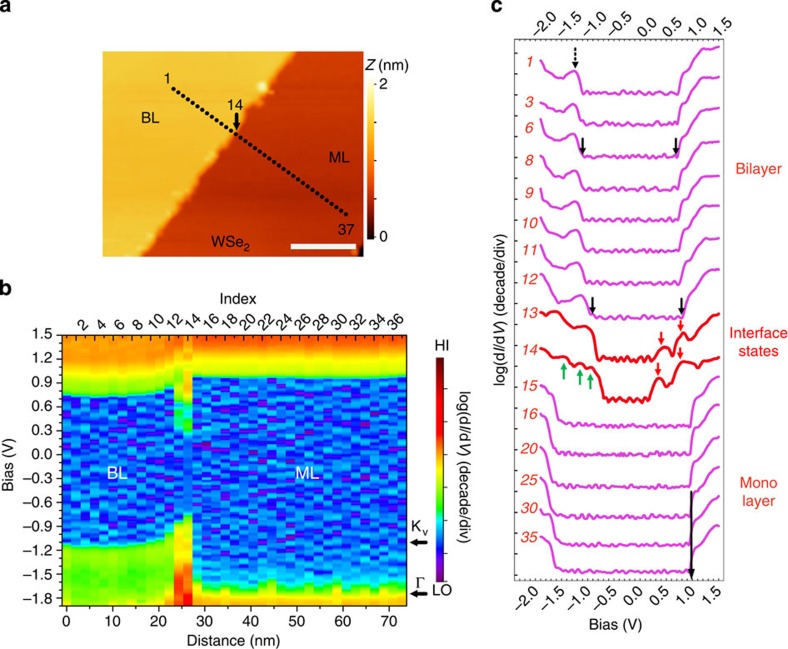
Scanning tunnelling spectroscopy investigation of band profile across the BL–ML WSe_2_ heterojunction. d*I*/d*V* spectra were taken along the path shown in (**a**). The spectra numbers are labelled (counting from left to the right in the path line). The total length is roughly 73 nm with a step size of 2 nm. Spectrum #14 was taken right at the interface. (**b**) Colour-coded rendering of the real space imaging of band profile plotted in terms of log(d*I*/d*V*). All spectra in this paper are displayed with arbitrary units (a.u.), except for Fig. 4c. The energy locations of Γ and K_V_ (actual VBM) points are labelled by black arrows. (**c**) A selective subset of log(d*I*/d*V*) spectra. In spectrum #1, the upper state of Γ splitting, which results from interlayer coupling, is labelled with a black dashed arrow. The interface states are marked in spectrum #13 and #14 with red arrows for conduction band side and green arrows for valence band side. A black arrow in monolayer region represents for the energy location of CBM. Scale bar, 20 nm (**a**).

The BL–ML lateral HJs are formed naturally when a second layer of TMD is grown on top of the ML TMD. We achieve direct growth of such a HJ either using CVD^27^ or molecular beam epitaxy (MBE)^26^,^28^. Shown in Fig. 1a is an example of a WSe_2_ BL–ML heterostructure grown on a highly oriented pyrolytic graphite (HOPG) substrate using CVD. In this STM image, three distinct regions—graphite, ML–WSe_2_ and BL–WSe_2_—are separated by two atomically sharp line interfaces, corresponding to the ML and BL step edges (respectively separating graphite from ML WSe_2_, and ML from BL WSe_2_). An atomically resolved STM image (Fig. 1b) taken on the BL region allows us to determine the orientation of these two interfaces to be along the zig-zag directions. Then scanning tunnelling spectra are acquired to reveal the real space band profile across the interface. Figure 2a is a close-up STM image showing the spatial locations where STS given in Fig. 2b,c is carried out. Shown in Fig. 2b is a colour rendering of the band mapping result, and selected individual spectra are shown in Fig. 2c. Note that d*I*/d*V* spectra are displayed on a logarithmic scale. The numbers on the spectra refer to their position in the complete set, in which spectra are acquired every 2 nm from the BL into the ML region, with spectrum #14 acquired at the interface (Fig. 2a).

The most striking feature in Fig. 2b is the apparent band bending for both conduction band and valence band in the BL WSe_2_ near the interface. The magnitude of the band bending Δ_bend_ is about 0.15 eV over about 10 nm towards the interface (depletion length). One can also see an abrupt change in the electronic structure right at the BL–ML interface (see also spectrum #13 and #14 in Fig. 2c). The spectra in the ML region, in contrast, exhibit little band bending.

In the BL region, the d*I*/d*V* spectrum shows a prominent peak in the valence band (labelled with black arrow in spectrum #1 of Fig. 2c). This corresponds to the higher energy branch at the Γ point split due to the interlayer coupling. This feature has been discussed in detail recently^29^. The lower state is not visible in the limited energy window here. Far away from the interface, the valence band maximum (VBM) of BL region is located at −1.10±0.07 eV while the conduction band minimum (CBM) is determined to be 0.71±0.07 eV (from spectrum #1–#6), corresponding to a quasiparticle bandgap of 1.81±0.10 eV. From #6 to #12, the spectral line shape remains the same but the locations of the VBM and CBM both move upward, corresponding to the band bending (as indicated by the arrows in #6 and #12).

Right at the BL–ML interface, new spectral features emerge (#13 and #14 in Fig. 2c). Two prominent peaks around 0.4 and 0.8 eV (marked by the red arrows) appear in the conduction band. The new spectral features in the valence band can be observed more vividly on spectrum #14 (marked by the green arrows) but merge with the bulk valence states in #13. Interestingly, as revealed by the STS, the interface states here behave effectively like a narrow-gap semiconductor with a gap value of 0.8 eV (with VBM and CBM located roughly at −0.6 to 0.2 eV, respectively). Our STS map (Fig. 2b) suggests that the interface states have type-I band alignment with 2D bulk states of both the BL and ML therefore can play the role of a quantum wire for both electrons and holes. Localized interface states are actually a common feature of semiconductor interfaces having broken bonds, such as surfaces states at crystal-vacuum interfaces. In our case, broken bonds occur at the termination of the upper layer of bilayer TMD. Thus we may expect the quantum wire states to be centred on the atoms terminating the edge. However, the details of the step structure are not yet fully known.

Moving into the ML WSe_2_ region, one quickly observes the bulk ML WSe_2_ electronic structure without visible band bending within the spatial resolution (2 nm) of the STS. As one can see from spectrum #15 to #30 the electronic structure remains constant. It is important to recognize that in the ML WSe_2_ region, it is difficult to directly observe the actual VBM location (at K point in Brillouin zone) using conventional constant *Z* tunnelling spectroscopy as in Fig. 2b,c, due to the extremely short tunnelling decay length of K-points states^29^. The peak in the valence band corresponds to the energy location of the Γ point while the VBM at K point (not visible in the constant *Z* spectroscopy) is approximately located at 0.65 eV above. This point will be discussed further below.

As in any HJ, the energy alignment of the band edges (including the interface quantum wire) right at the interface is an inherent property, while the band bending depends on the electrostatics (for example the substrate). In the bulk ML and BL regions, the intimate contact with the graphite determines the location of *E*_F_ in the gap. Near the edge, band bending occurs in order to keep the *E*_F_ in the bandgap of the zig-zag quantum wire. The screening by the substrate graphite sets the length scale of the band bending at about 10 nm.

### MoSe_2_ bilayer–monolayer HJs

**Figure 3:**
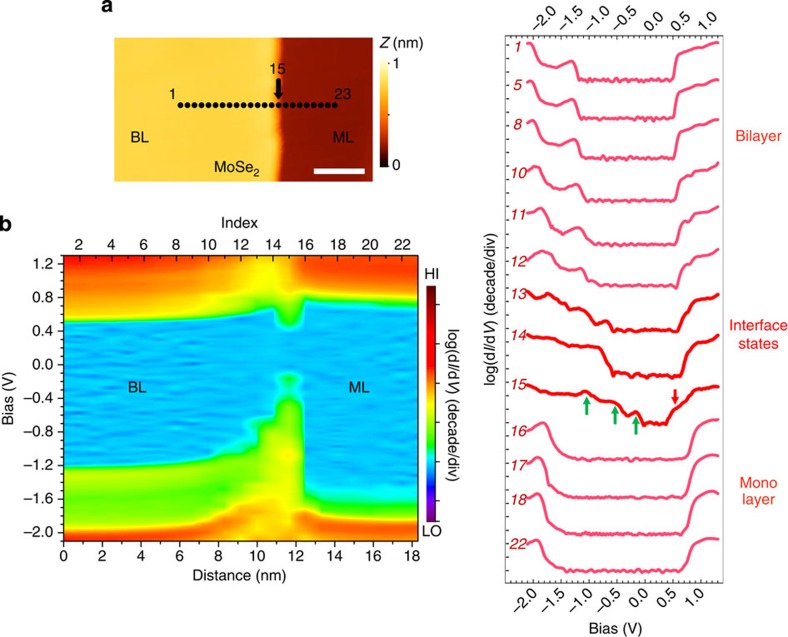
Scanning tunnelling spectroscopy investigation of band profile across the BL–ML MoSe_2_ heterojunction. Similar with Fig. 2, (**a**) is a close-up STM image showing the path line that spectroscopy was taken along. The total length is about 18 nm with a step size of 0.8 nm. The spectra numbers in **b** and **c** are counted from left to right in the path, while spectrum #15 was taken right at the BL–ML interface. (**b**) Colour-coded rendering of the real space imaging of band profile plotted in terms of log(d*I*/d*V*). (**c**) Selective subset of log(d*I*/d*V*) spectra. In spectrum #15, the interface states in valence band and conduction band are marked with green and red arrows, respectively. Scale bar, 5 nm (**a**).

We have also studied the BL–ML heterostructure in MBE-grown MoSe_2_ on HOPG, where we find the same general behaviour as for WSe_2_, as illustrated in Fig. 3. The spatially resolved STS is acquired with a finer step of 0.8 nm (labelled in Fig. 3a). The colour rendering of band mapping based on the STS is shown in Fig. 3b. The upward band bending is observed for both conduction and valence bands. However, the ‘apparent' band bending from the valence band side is much stronger, effectively reducing the bandgap starting about 3–4 nm away from the edge. On detailed inspection, this is due to the overlap of valence band states of the bulk with those of the interface quantum wire states in the STS signal. One can see this more clearly from the individual STS spectra shown in Fig. 3c. Spectrum #10 still resembles the bulk BL band structure. On the other hand, in spectrum #11 the interface states above the bulk valence band edge start emerging and eventually split off (labelled by the green arrows in spectrum #15). This behaviour is similar to that in WSe_2_ discussed above except here the magnitude of the energy split off from the VBM of the bilayer is larger. The conduction band interface state is only observed right at the interface (#15, labelled by the red arrow). This might be due to a smaller energy split off from the CBM of the bilayer making it only observable very near the edge. The edge here has a narrower gap of 0.4 eV, which is also smaller than the one in WSe_2_. Moreover, with finer spatial resolution, we can observe a small band bending in ML region with a very short depletion length (around 1 nm only).

**Figure 4:**
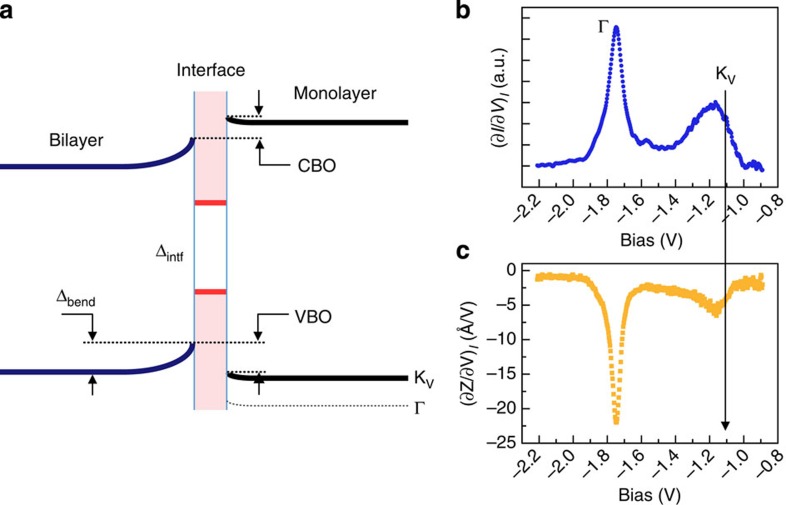
Schematic diagram of band alignments and constant *Z* spectroscopy for valence band of ML WSe_2_. (**a**) Schematic diagram showing the generic band alignment in BL–ML TMDs HJs. The magnitude of the band bending is labelled as Δ_bend_, and the gap of interface states is Δ_intf_. (**b**,**c**) Two modes of constant *Z* spectroscopy—(∂*I*/∂*V*)_*I*_ and (∂*Z*/∂*V*)_*I*_ for valence band of monolayer WSe_2_. The actual valence band maximum at K point is labelled as K_V_, which is about 0.65 eV above Γ point. See ref. 29 for details.

We summarize the general behaviour of lateral HJs formed from BL and ML WSe_2_ or MoSe_2_ with the schematic diagram (Fig. 4a) and Table 1. Here, the amount of the energy band bending is labelled as Δ_bend_, the gap of the interface mode as Δ_intf_, the valence band offset as VBO and the conduction band offset as CBO. The numerical values for these quantities are shown in Table 1 for WSe_2_ and MoSe_2_, respectively. As mentioned above, in the ML region, constant height tunnelling spectroscopy lacks the sensitivity to reveal the location of the VBM at the K point. On the other hand, as we reported recently^29^, constant-current spectroscopy can overcome this difficulty and resolve the states at the K point in the valence band (labelled as K_V_). As discussed extensively in ref. 29, in such constant-current spectroscopy, the individual thresholds at different critical points appear as peaks in differential conductivity (∂*I*/∂*V*)_*I*_ as well as dips in the derivative of the tip-to-sample position as a function of the voltage (∂*Z*/∂*V*)_*I*_. Shown in Fig. 4b and c is the case for WSe_2_. The actual VBM is located at the mid-point of the transition from the TMD to the graphite states, which is around 0.65 eV above the Γ point, as labelled in Fig. 2b. For MoSe_2_, this value is 0.40±0.04 eV. With this information, we are able to determine that the CBO and VBO of BL–ML WSe_2_ HJs are 0.15±0.10 and 0.12±0.10 eV, respectively, as shown in Table 1. Similarly, for BL–ML MoSe_2_, they are found to be 0.08±0.10 and 0.43±0.10 eV, respectively, albeit in this case, the determined CBO is smaller than the experimental uncertainty of 0.1 eV.

## Discussion

We first discuss the implications for electron transport across or along the interface which raises many interesting issues. It is well known for semiconductor HJs that there can be strong reflection at the interface, depending on how dissimilar the two material are. The same is true for reduced-dimensionality interfaces, such as HJs between ML and BL graphene^30^,^31^. Considering the significant differences of their electronic structures (especially band edges located at different **k** points in Brillouin zone), we may expect similar strong reflection at the interfaces of these BL–ML TMDs HJs. Transport along the interface can also be subject to scattering by imperfections. For example, the edge of the upper layer is in general not perfectly straight, so transport along the quantum wire will experience some disruption at kinks and other imperfections in the edge structure. This could lead to hopping conductivity with poor mobility along the wire. However, the non-ideal transport need not be an obstacle to important applications. In many conventional applications, the primary role of the HJ is to provide confinement, or to control barriers; and transport along or across the interface is secondary or even irrelevant. More important is that these interfaces provide a rich combination of barriers and localized states, offering novel opportunities for device engineering. Moreover we anticipate that the localized quantum wire states could be easily doped, since atoms tend to diffuse and bind at step edges.

In conclusion, our STM/STS studies reveal a new class of lateral HJ in atomically thin WSe_2_ and MoSe_2_, formed from naturally existing BL–ML thickness terraces with zig-zag orientation. Because of the thickness dependence of the bandgap and band edges, the ML and BL bulk bands align to form a type-I HJ, as clearly seen in our STS maps. The interface states in both systems form a narrow-gap quantum wire. This interface quantum wire has a gap value of 0.8 eV in WSe_2_ and a smaller gap value of 0.4 eV in MoSe_2_. The band alignment of the quantum wire states is also type-I with respect to both the BL and ML sides of the junctions. We expect that the unique properties of this novel class of HJs will create new possibilities for device applications based on 2D TMDs.

## Methods

### Growth of 2D TMDs samples

The preparation of WSe_2_ crystal flakes by the vapour-phase reaction has been reported before^27^. In brief, high purity metal trioxides WO_3_ was placed in a ceramic boat in the centre of a furnace, while graphite substrate was placed in the downstream side of the furnace, adjacent to the ceramic boat. Selenium powder was heated by a heating tape and carried by Ar or Ar/H_2_ gas to the furnace heating centre. The temperature of furnace was gradually raised from room temperature to the desired temperature, and cooled down naturally after the reaction had occurred. MoSe_2_ was grown on freshly cleaved HOPG substrate using MBE in an ultra-high vacuum (UHV) chamber which has a base pressure of 5 × 10^−11^ torr. High-purity Mo (99.95%) and Se (99.999%) were evaporated from a home-built e-beam evaporator and an effusion cell, respectively, with a ratio of 1:30. The graphite substrate was kept at 550 °C, and the growth rate was about 0.3 layer per hour. The sample was annealed in a Se flux at 600 °C for 30 min after growth. Before STM studies, the CVD samples are cleaned in the UHV chamber (base pressure is <6 × 10^−11^  torr) by annealing the sample at 300 °C for 6 h. The MBE samples are transferred *in situ* between the growth chamber and the STM chamber under UHV environment.

### Scanning tunnelling microscopy and spectroscopy

All STM investigations reported here were acquired at 77 K in UHV (base pressure is <6 × 10^−11^  torr). Electrochemically etched tungsten tips were cleaned *in situ* with electron beam bombardment. The tunnelling bias is applied to the sample. The conductance spectra were taken by using a lock-in amplifier with a modulation voltage of 10 mV and at a frequency of 924 Hz.

## Additional information

**How to cite this article:** Zhang, C. *et al*. Visualizing band offsets and edge states in bilayer-monolayer transition metal dichalcogenides lateral heterojunction. *Nat. Commun.* 7:10349 doi: 10.1038/ncomms10349 (2016).

## Figures and Tables

**Table 1:** Band alignments in bilayer-monolayer heterojunctions for WSe_2_ and MoSe_2_.

**BL**–**ML**	**Δ**_**bend**_ **(BL)**	**CBO**	**VBO**	**Δ**_**intf**_
WSe_2_	0.15±0.05 eV	0.15±0.10 eV	0.12±0.10 eV	0.8±0.10 eV
MoSe_2_		0.08±0.10 eV	0.43±0.10 eV	0.4±0.10 eV

BL, bilayer; CBO, conduction band offset; ML, monolayer; VBO, valence band offset.

The s.d.'s shown here are based on statistics of multiple measurements (more than 50 times).
